# Novel Lineage of Infectious Bronchitis Virus from Sub-Saharan Africa Identified by Random Amplification and Next-Generation Sequencing of Viral Genome

**DOI:** 10.3390/life12040475

**Published:** 2022-03-25

**Authors:** Krisztina Bali, Eszter Kaszab, Szilvia Marton, Seydou Hamadou Hamdiou, Reza Karim Bentaleb, István Kiss, Vilmos Palya, Krisztián Bányai

**Affiliations:** 1 Veterinary Medical Research Institute, 1143 Budapest, Hungary; kaszab.eszter@vmri.hu (E.K.); martonsil@gmail.com (S.M.); 2 Ceva Sante Animale S.A, 33500 Libourne, France; seydouhxh@gmail.com (S.H.H.); reza.bentaleb@ceva.com (R.K.B.); 3 Ceva-Phylaxia Veterinary Biologicals Co., Ltd., 1107 Budapest, Hungary; istvan.kiss@ceva.com (I.K.); vilmos.palya@ceva.com (V.P.); 4 Department of Pharmacology and Toxicology, University of Veterinary Medicine, 1078 Budapest, Hungary

**Keywords:** avian coronavirus, phylogenetic analysis, whole-genome sequencing, Ivory Coast, Ghana, Cameroon

## Abstract

Avian infectious bronchitis (IB) is among the major viral respiratory and reproductive diseases of chickens caused by Avian coronavirus. In the African continent, IB was first described in countries located in the Mediterranean basin. In other parts of the continent, the epidemiological situation of IB remains unclear. In this study, the complete genome sequences of five IBV strains, originating from the sub-Saharan area were determined. Phylogenetic analysis based on the full-length S1 sequences identified three lineages (GI-14, GI-16, and GI-19) common in Africa and revealed that a strain, D2334/11/2/13/CI, isolated in Ivory Coast may represent a novel lineage within genotype GI. The maximum inter- and intragenotype sequence identities between this strain and other IBVs were 67.58% and 78.84% (nucleotide) and 64.44% and 78.6% (amino acid), respectively. The whole-genome nucleotide identity of the novel variant shared the highest values with a reference Belgian nephropathogenic strain (B1648, 92.4%) and with another study strain from Ivory Coast (D2334/12/2/13/CI, 94.6%). This study illustrates the importance of epidemiological monitoring of IBV in sub-Saharan Africa, as the area may serve as a focal point for newly emerging viral lineages.

## 1. Introduction

Infectious bronchitis (IB) is a highly contagious viral disease that affects the respiratory, reproductive, and renal systems of chickens of all ages and types [1]. Infectious bronchitis was first described in the 1930s in the USA [2,3] and has since been reported from numerous countries worldwide [4,5,6,7,8,9]. The causative agent, infectious bronchitis virus (IBV), belongs to the species *Avian coronavirus* (ACoV), genus *Gammacoronavirus*, family *Coronaviridae* [10]. IBV is an enveloped, positive-sense, single-stranded RNA virus with a 27 kb genome. The general genome organization of ACoV is 1ab-S-3a-3b-E-M-4b-4c-5a-5b-N-6b [11]. The genome contains 13 open reading frames that code for 25 proteins. The key protein containing neutralization epitopes is the spike protein that is post-translationally cleaved into two subunits, S1 and S2. The S1 is responsible for the attachment of the virus to the cellular membrane and plays a major role in tissue tropism, induction of protective immunity, virus neutralization, and serotype specificity [12]. The S1 gene sequencing is the most widely used method for classifying IBV isolates into genotypes. According to the S1 gene-based classification system, there are 8 genotypes (GI to GVIII) and 37 lineages [13]. The high mutation and recombination rates have led to the emergence of new variants, including lineage GI-28, GI-29 as well as genotype GVII in intensive poultry farms in China, GVIII in Poland, and recently, GI-30 in Trinidad and Tobago [14,15,16,17,18].

The impact of IB on poultry in the Sub-Saharan region of Africa is still poorly known [19]. Nonetheless, reports have shown that IBV is common in poultry flocks with respiratory disease and a severe drop in egg production [20]. IB is controlled mainly by vaccination in many African countries, using either live-attenuated or inactivated vaccines [21]. In this region, the first documentation of IB originates from Nigeria [22,23]. Subsequent reports that relied on serological assays showed evidence of widespread distribution of IBV in Nigeria. Owoade and coworkers have shown a seroprevalence rate as high as 84% for IBV [24]. Other studies from this area have also reported high levels of circulation of IBV in backyard poultry and in commercial poultry farms with seroprevalence rates above 70% [24,25,26,27]. The exposure of captive wild birds and indigenous local chickens is significant, with a measured seroprevalence of 11.6% and 49.1%, respectively [28]. Furthermore, there is serological evidence of co-circulation of multiple IBV antigenic types [20]. In West Africa, only a few studies have examined the prevalence of IBV with molecular methods [20,27,29,30]. In Burkina Faso, the prevalence of IBV was 3.9% by using RT-PCR [29]. A study from Ghana revealed that 40% of the samples tested positive with PCR for IBV and 20% of the samples were found to contain both IBV and Newcastle disease virus [30]. Although reports show that IBV may play a significant role in respiratory disease outbreaks in poultry in Ghana, vaccination against IBV is not carried out [31]. In Ivory Coast, the presence of IB in commercial layer farms is reported on a regular basis based on clinical signs (respiratory signs, decline of the egg production), but IBV is also associated with subclinical infections in backyard poultry. Both seroprevalence (72.3%) and PCR positivity (14.6%) are considered high. Vaccination is strongly recommended, mainly based on the M41 strain, although there has been no prior study of the circulation of IBV in the country [19]. Overall, published records indicate that IBVs circulate in this neglected area of Africa, but a significant shortcoming is that it is hard, if not impossible, to distinguish between the immune response raised by natural infection and vaccination, as well as between positive PCR results originating from infection with a field strain and those associated with shedding vaccine strains. Thus, the true economic burden associated with IB remains unknown.

At present, there are only limited molecular data on IBV strains from the sub-Saharan region of Africa. Most of the information is from a recent study performed in Nigeria and Niger. In the GenBank database, roughly three dozen full-length spike 1 (S1) gene sequences and a single full-length genome sequence are available [32]. One of these S1 sequences was assigned to the widely distributed GI-1 lineage, while the remainder sequences belong to the GI-12 and GI-26 lineages, the latter representing the majority of strains with available sequence information (*n* = 32). Until now, GI-12 strains (D274-like) were only reported in Europe and Africa, whereas the lineage GI-26 represents a unique African group of IBVs isolated in Niger and Nigeria [32,33,34].

The present study gives further insight into the genetic diversity of IBV in Africa using archived samples. The study shows that in addition to the common lineages indigenous to the region, Africa may also serve as a focal point of emerging new variants, a finding that warrants more intensive surveillance on IB.

## 2. Materials and Methods

### 2.1. Virus Isolates

During 2013, as part of a surveillance program, samples from various industrial flocks located in Cameroon, Ghana, and Ivory Coast were sent to the diagnostic laboratory of CEVA-Phylaxia (Budapest, Hungary) to detect IBV. Molecular characterization of five strains isolated on embryonated eggs was performed in 2020 when the strains were shared with collaborating partners at the Veterinary Medical Research Institute. All isolates originated from broiler-type chicken (aged 27 to 60 days) with various clinical and pathology backgrounds (Table 1).

### 2.2. Whole-Genome Sequencing

Viral isolates were passed through a 0.45 µm sterile filter (Nantong FilterBio Membrane Co., Ltd., Nantong, China). Next, a mixture of nucleases was added to the sample to eliminate free nucleic acids that could have interfered with the output of sequencing. These steps of viral RNA enrichment were followed by extraction of viral RNA and then sequence-independent amplification by using a random primed RT-PCR protocol. The random amplified DNA was analyzed by electrophoresis in 1% agarose gel stained with GelRed (Biotium, Hayward, CA, USA) and the obtained smear was excised and extracted from the gel using the Gel/PCR DNA Fragments Extraction Kit (Geneaid Biotech Ltd., Taipei, Taiwan). Nucleotide sequences were determined by next-generation sequencing on an Ion Torrent PGM (Life Technologies/Thermo Fisher Scientific, Waltham, MA, USA) platform following the protocols described previously [35,36].

### 2.3. Sequence Data and Analysis Tools

The sequencing reads were trimmed based on quality and they were de novo assembled into contigs by using Geneious Prime (Biomatters Ltd., Auckland, New Zealand). Using the obtained contigs of various sizes, a BLAST (National Center for Biotechnology Information, Bethesda) search against a virus database was performed to identify best-matching IBV genomic sequences. Next, the reads were mapped to the available reference sequences, and then, the consensus sequence was extracted. Reference S1 gene as well as whole-genome sequences of IBV strains, including those from West Africa and Central Africa, isolates were downloaded for comparison from the NCBI’s nucleotide sequence database (https://www.ncbi.nlm.nih.gov/, accessed on 1 September 2021). The complete consensus genomic sequences were deposited in the GenBank, with the accession numbers, MZ325296 to MZ325300.

The prediction of open reading frames (ORF) was carried out using the ‘Find ORFs’ module implemented in Geneious Prime and the ORF-finder program (https://www.ncbi.nlm.nih.gov/orffinder/, accessed on 1 August 2021). Multiple alignments were prepared using the MAFFT algorithm in the Geneious Prime program. The full S1 sequences determined in this study were aligned together with reference sequences representing 32 lineages within 6 genotypes and 26 unique variants, as recommended by Valastro and coworkers [13]. Additionally, sequences representing the three newly identified GI-28, GI-29, and GI-30 lineages and the two GVII and GVIII genotypes were also included in the analyses [14,15,16,17].

A phylogenetic tree based on the S1 gene was constructed using the maximum likelihood (ML) method with the general time-reversible (GTR) nucleotide substitution model with a discrete gamma distribution (+G), assuming that a certain fraction of sites are evolutionary invariable (+I) with 1000 bootstrap replicates in MEGA X. [37]. The percentages of nucleotide and amino acid identities were obtained from pairwise distances calculated in MEGA X with the *p*-distance method. A heatmap was generated with the use of the Clustvis web tool (https://biit.cs.ut.ee/clustvis/, accessed on 15 September 2021) according to Metsalu and Vilo (2015) [38].

Possible recombination events were screened by using the recombination detection program 4 (RDP4, Version 4.97) [39]. A total of 100 complete genome sequences showing the highest sequence similarity based on the BLAST search were downloaded from the GenBank to perform this analysis. Furthermore, complete genome sequences of 12 vaccine strains were selected according to the vaccination history of birds. To obtain a conservative estimate, a recombination event would only be accepted if it was detected by five or more methods implemented in the program, with a *p*-value less than 5 × 10^−4^. The putative recombination events were further analyzed by using the SimPlot software (Version 3.5.1) [40].

## 3. Results

The output of next-generation sequencing runs is depicted in Table 2. The length of complete-genomes of the five study strains without the poly-A tail varied between 27,616 and 27,671 nucleotides (nt).

The order of ORFs between the 5′ and 3′ untranslated regions (UTRs) was conserved (5′-UTR-1a-1ab-S-3a-3b-E-M-4b-4c-5a-5b-N-6b-3′UTR). The two large polyproteins, 1a and 1ab, are proteolytically processed following translation to yield 15 functional proteins (nsp2–nsp16). Variation in sequence length was seen in some ORFs (Table 3). Variable genomic regions included ORF1a (range 11,829 to 11,862 nt), ORF1ab (19,863 to 19,896 nt), spike protein gene (3498 to 3507 nt), ORF 3b (186 to 195 nt), envelope and membrane protein genes (282–324 nt and 672–678 nt, respectively), ORF4b (201–285 nt), nucleocapsid protein gene (1224–1230 nt) and ORF6b (222–225 nt). The ORF3a (174 nt), ORF4c (171 nt) ORF5a (198 nt), and ORF5b (249) did not show a variation in sequence length.

**Figure 1:**
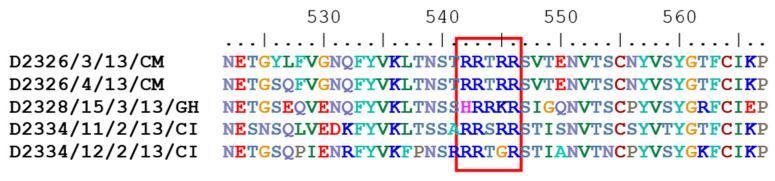
Amino acid sequences of S1/S2 cleavage sites of IBV strains isolated in this study. Amino acids are shown as single characters, the cleavage site motif is highlighted in the red frame.

The five study strains were also diverse in the deduced amino acid sequence of the cleavage recognition site motif within the spike protein precursor (Figure 1). The S1 cleavage site of the strains from Cameroon (D2326/3/13/CM/2013 and D2326/4/13/CM/2013) was Arg-Arg-Thr-Arg-Arg (R-R-T-R-R). The putative S1 cleavage site motifs of the Ghanaian strain, D2328/15/3/13/GH/2013, and the two strains from Ivory Coast, D2334/12/2/13/CI/2013 and D2334/11/2/13/CI, were His-Arg-Arg-Lys-Arg (H-R-R-K-R), Arg-Arg-Thr-Gly-Arg (R-R-T-G-R) and Arg-Arg-Ser-Arg-Arg (R-R-S-R-R), respectively.

**Figure 2:**
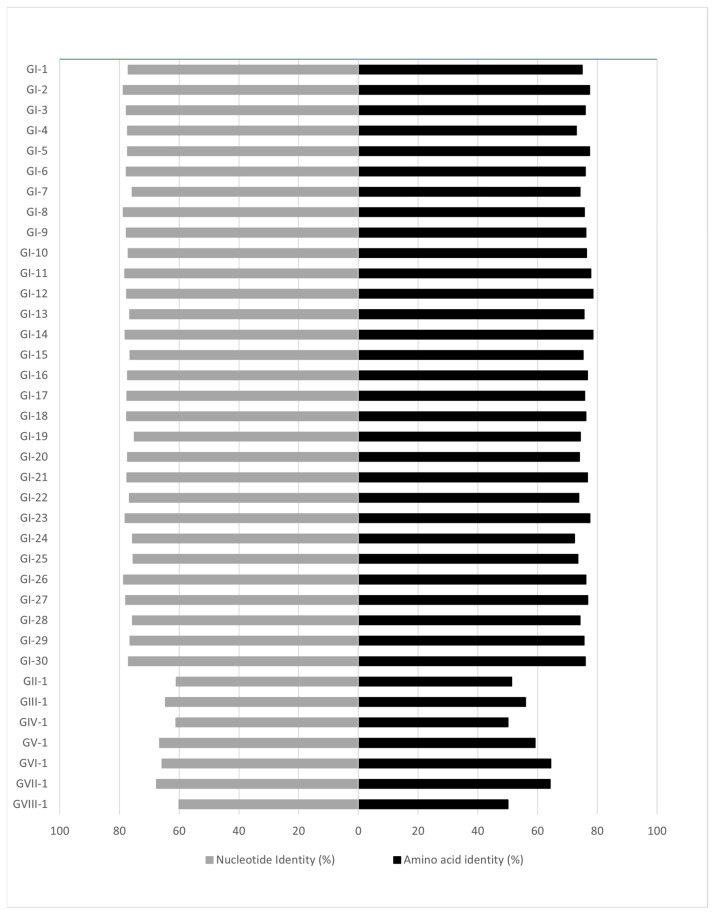
Pairwise nucleotide and amino acid identities of the S1 genomic region between the novel GI-31 lineage and the reference strains according to Valastro and coworkers [13].

**Figure 3:**
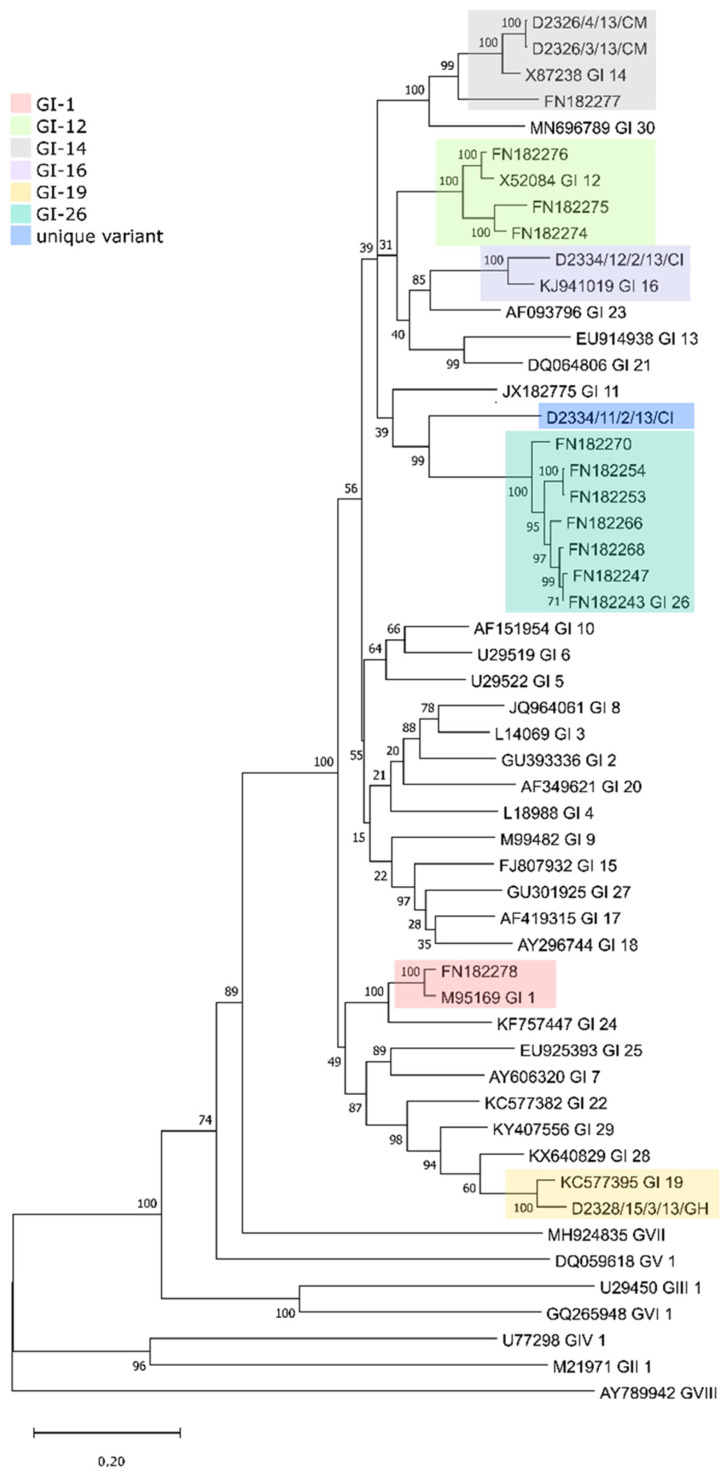
Phylogenetic analysis of the full-length IBV S1 genes from the West African strains and 35 reference strains, each representing a particular lineage. The tree was constructed with the maximum likelihood method with the general time-reversible nucleotide substitution model and 1000 bootstrap replicates.

The phylogenetic classification based on the spike 1 (S1) protein-coding region [13] identified three genotypes and a unique variant among the five study strains, including two GI-14 strains (B1648-like; D2326/3/13/CM, D2326/4/13/CM) from Cameroon, one GI-19 (QX-like; D2328/15/3/13/GH) from Ghana and one GI-16 strain (Q1-like; D2334/12/2/13/CI) and a novel lineage (D2334/11/2/13/CI) from Ivory Coast. The phylogenetic tree based on the S1 gene demonstrates that the D2334/11/2/13/CI strain differs from strains belonging to other lineages and genotypes (maximum inter- and intragenotype identities, nt, 67.58% and 78.84%, aa, 64.44%, and 78.6%, respectively; Figure 2 and Figure 3, Tables S1 and S2). Furthermore, this unique strain branched separately from the Nigerian GI-26 reference strain (nt and aa identities, 78.84%, and 76.24%, respectively; Figure 3). Additional analyses failed to identify any recombination event when comparing D2334/11/2/13/CI with a representative number (*n* = 112) of reference strains (data not shown). Hence, we classified this strain as the first member of a new lineage, designated tentatively as GI-31.

To further investigate the genetic relationship among the study strains with a focus on the novel variant, D2334/11/2/13/CI, other genomic regions were also analyzed. The genome-wide pairwise nucleotide identity of this unique variant with 27 reference sequences collected from the GenBank ranged between 85.9% and 92.4%, sharing the highest nucleotide identity with a Belgian strain, B1648. The nucleotide identity compared to the single complete genome sequence available from West Africa (NGA/A116E7/2006, lineage GI-26) was 91.3%. The unique variant, D2334/11/2/13/CI, showed the highest complete genome sequence identity (94.6%) with another study strain from Ivory Coast (D2334/12/2/13/CI). An overview of genome-wide nucleotide identity values among the five study strains is shown in Table 4. The genome-based identity among the remaining African strains originating from other regions ranged from 91.3% to 88.3%.

**Figure 4:**
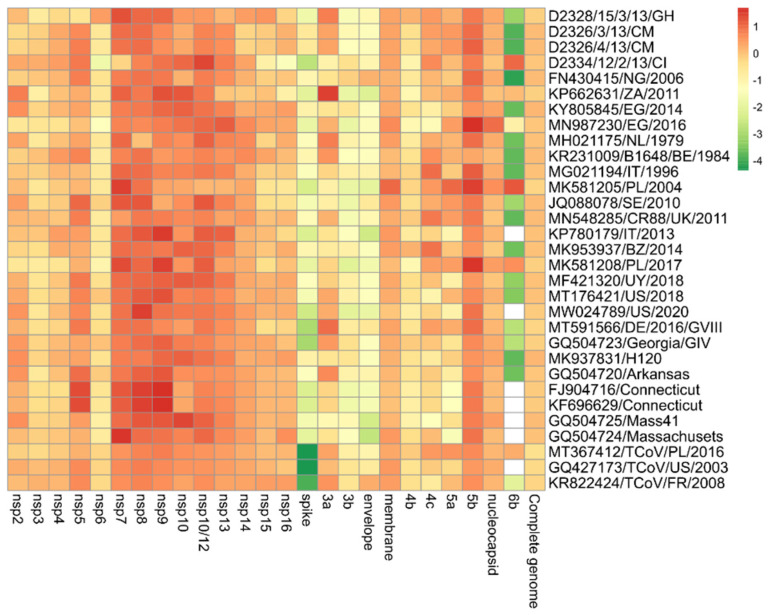
Heatmap showing the relationship of IBV isolate D2334/11/2/13/CI on the basis of pairwise distances for each gene in relation to other viruses. The most closely related genes have a deep red color that converts to orange, yellow, and deep green with increasing distance. White boxes indicate that no 6b protein is encoded.

The pairwise nucleotide comparisons between the 13 ORFs and the 15 nsps of the unique variant with the newly assigned 4 IBV isolates and the other 27 strains obtained from the GenBank database are depicted in Figure 4. The unique variant strain shared the highest nucleotide identity with the other Ivorian strain (D2334/12/2/13/CI) in the case of seven out of thirteen (7/13) ORFs (ORF 3b, 4c, 5a, 5b, 6b, membrane, and nucleocapsid protein genes) and nine out of fifteen (9/15) nsps (nsp2-nsp5, nsp9-nsp14). Four nsps (nsp6-nsp8, nsp15) and the ORF 4b shared the greatest sequence identity with the newly determined Ghanaian strain (D2328/15/3/13/GH). The nsp16 of the novel lineage was the most identical (93.4%) to a TCoV strain (KR822424/TCoV/FR/2008). ORF 3a was most similar (97.1%) to another African strain (KP662631/ZA/2011). The envelope protein-coding gene was mostly identical (92%) to the Nigerian strain (FN430415/NG/2006). In the similarity matrix, we added some turkey origin coronaviruses (TCoVs) because BLAST analyses showed a high degree of sequence identity between study strains and TCoV strains in the replicase complex (nsp2-nsp16), membrane (M), 5b and nucleocapsid (N) genes with a >90% (up to 96.9%) sequence identity for each (Figure 4) [41,42,43].

## 4. Discussion

The aim of the present study was to characterize IBV strains originating from Western and Central Africa. IB is among the major viral respiratory and reproductive diseases of chickens affecting poultry production in parts of Africa. Yet, the epidemiological situation of IBV, particularly in the tropical countries of the sub-Saharan region, is still unexplored due to a lack of optimized surveillance programs and the absence of adequate sequence data from the circulating strains. The availability of study strains from a single year, 2013, illustrates well the lack of surveillance efforts.

The majority of information on the genetic diversity of IBV variants circulating in Africa comes from North African countries, such as Egypt, Morocco, and Tunisia, where besides the widely distributed GI-1 (Mass, H120), GI-13 (4/91, CR88, 793B), GI-16 (Q1-like) and GI-19 (QX-like) strains, GI-12 (D274-like), GI-21 (Italy 02) and GI-23 (Variant 2) strains were also reported [44,45,46,47,48,49,50,51]. In other parts of the continent, data on IBV strain diversity is scarce. Nonetheless, GI-13 and GI-19 IBV strains were detected lately in Algeria, GI-19 in Ghana, and some IBV variants were reported in recent years from Libya and Ethiopia [52,53,54,55,56]. Strains belonging to GI-12, GI-14, GI-16, GI-19, GI-23, and GI-26 lineages were isolated from Nigeria [20, unpublished GenBank records]. Additionally, studies reported the circulation of lineages GI-1, GI-13, and GI-19 from South Africa and Zimbabwe [48,57]. Our study extends the knowledge of circulating IBV strains, even if both temporal and spatial representation of study strains were limited. We identified lineage GI-14 strains from Cameroon, a GI-16 from Ivory Coast, and a GI-19 strain from Ghana as well as a putative novel lineage from Ivory Coast, a genetic variant that can be differentiated from other lineages by sequence identity and phylogenetic analyses.

The S1 protein, being responsible for the attachment to the host cells is involved in tissue tropism, pathogenicity, and virus neutralization; yet, the association between viral genetic features and clinical disease has not been unequivocally demonstrated for IBV [58]. Analyses of the S1 protein showed that the primary structure of its cleavage recognition site is not associated with the genotype or serotype specificity. Moreover, it seems to be irrelevant to the viral pathogenicity and tissue tropism, although this region of the protein shows a continuous evolution in various IBV strains [58,59]. In this study, four sequence variants of the S1 protein cleavage recognition motif were found among the five isolates. The S1 cleavage site motif of GI-14 strains from Cameroon (D2326/3/13/CM/2013 and D2326/4/13/CM/2013) was shared with the motif of lineage GI-23 strains from Egypt, Iran, and Poland [34,60,61,62]. The S1 cleavage site motifs of the Ghanaian GI-19 strain (D2328/15/3/13/GH/2013) and the GI-16 strain from Ivory Coast (D2334/12/2/13/CI/2013) were commonly observed in other isolates with shared genetic lineages [63,64]. The unique strain isolated in Ivory Coast (D2334/11/2/13/CI) has a cleavage recognition site of Arg-Arg-Ser-Arg-Arg (R-R-S-R-R), and might be an ancient and common cleavage site motif that was observed in numerous IBV genotypes and serotypes and even in other coronaviruses (such as alpha-, beta-, and gammacoronaviruses) [58,62,65]. The S1 gene serves as the basis of subgenotype classification with 29 published lineages and at least 26 unique variants within genotype GI IBVs. It is clear that multiple S1-gene-based lineages of genotype GI IBVs may cause a particular disease and the same clinical signs may be caused by different lineages. Regarding the widely distributed African lineages, in previous reports, genotype GI-14 strains were characterized as being nephropathogenic and causing egg production problems [66]. Lineage GI-16 was linked to respiratory syndrome, nephropathogenic disease, and severe drops in egg production [32,67,68,69]. GI-19 IBV strains were associated with respiratory and intestinal signs as well as with disorders of the urinary and reproductive tracts [70,71,72,73]. All study strains from West and Central African countries, including the tentative lineage GI-31 strain, were isolated from flocks showing respiratory disease with or without diarrhea. The question of why a particular clinical manifestation dominates in IB outbreaks seems to be a complex question and, in addition to the viral genotypes, it may involve host immunologic and genetic factors that need to be determined.

In summary, this survey demonstrates that common IBV lineages indigenous to Central and Western Africa circulated in the mid-2010s and showed that the study region may serve as a focal point of emerging new variants, a finding that warrants more intensive surveillance on IB in the tropical countries of Africa.

## Figures and Tables

**Table 1:** Characteristics of infectious bronchitis virus (IBV) isolates whose genome sequence was determined in this study.

Isolate	Age (d)	Organ of Isolation	Country of Origin	Clinical Features/Pathology	Vaccination History against IBV	Genotype of Study Strain
D2326/3/13/CM/2013	42	I	Cameroon	respiratory signs, diarrhea	not vaccinated	GI-14 (B1648-like)
D2326/4/13/CM/2013	60	I	Cameroon	respiratory signs: groans, snoring, mucoid nasal discharge	not vaccinated	GI-14 (B1648-like)
D2328/15/3/13/GH/2013	27	CT	Ghana	respiratory signs: hard breathing, groans, nasal discharges, digestive signs: green feces	not vaccinated	GI-19 (QX-like)
D2334/11/2/13/CI/2013	31	CT	Ivory Coast	nasal discharge, groans, diarrhea, growth problem	D1: H120	unique variant (GI-31)
D2334/12/2/13/CI/2013	45	CT	Ivory Coast	respiratory signs, diarrhea	D1: H120, D28: Mass	GI-16 (Q1-like)

CT—caecal tonsil; I—intestine.

**Table 2:** Sequence length of ORFs.

Study Strains	All Read Output	Assembled Reads	Assembled Nucleotides	Mean Sequencing Depth (X)	Genome Size *
D2326/3/13/CM/2013	40,932	33,342	5,960,515	205	27,641
D2326/4/13/CM/2013	40,949	31,821	5,818,590	180	27,671
D2328/15/3/13/GH/2013	77,472	62,435	12,043,754	391	27,616
D2334/11/2/13/CI/2013	97,955	80,170	17,037,995	559	27,640
D2334/12/2/13/CI/2013	74,763	56,316	11,871,797	456	27,646

* Without poly-A tail.

**Table 3:** Sequence length of ORFs.

	ORF Length (nt)												
Study Strains	1a	1ab	Spike	3a	3b	Envelope	Membrane	4b	4c	5a	5b	Nucleocapsid	6b
D2326/3/13/CM/2013	11,841	19,875	3498	174	186	324	672	285	171	198	249	1230	225
D2326/4/13/CM/2013	11,862	19,896	3501	174	192	324	672	285	171	198	249	1230	225
D2328/15/3/13/GH/2013	11,829	19,863	3498	174	192	285	678	285	171	198	249	1224	225
D2334/11/2/13/CI/2013	11,850	19,884	3507	174	195	282	672	201	171	198	249	1230	222
D2334/12/2/13/CI/2013	11,862	19,896	3501	174	195	282	672	285	171	198	249	1230	222

**Table 4:** Complete genome nucleotide identity matrix of study strains.

	D2334/11/2/13/CI	D2334/12/2/13/CI	D2326/4/13/CM	D2326/3/13/CM	D2328/15/3/13/GH
D2334/11/2/13/CI					
D2334/12/2/13/CI	94.58				
D2326/4/13/CM	91.42	91.62			
D2326/3/13/CM	91.35	91.55	99.37		
D2328/15/3/13/GH	90.23	90.05	89.15	89.12	

## Data Availability

Sequence data were deposited in GenBank (accession no.: MZ325296-MZ325300).

## Supplementary Materials

The following supporting information can be downloaded at: https://www.mdpi.com/article/10.3390/life12040475/s1, Table S1: Pairwise nucleotide identity matrix of the S1 genomic region between the reference strains; Table S2: Pairwise amino acid identity matrix of the S1 genomic region between the reference strains.
