# Supplementary material for: Novel Lineage of Infectious Bronchitis Virus from Sub-Saharan Africa Identified by Random Amplification and Next-Generation Sequencing of Viral Genome
Source: Life (Basel). 2022 Mar 25;12(4):475. doi: 10.3390/life12040475 (PMC9028189; doi:10.3390/life12040475)
Supplement: Supplementary file 1 [file life-12-00475-s001.zip › life-1640557-supplementary.pdf]

life

Supplementary Material of Novel Lineage of Infectious Bronchitis Virus from Sub-Saharan Africa Identified by Random Amplification and Next-Generation Sequencing of Viral Genome

Table S1. Pairwise nucleotide identity matrix of the S1 genomic region between the reference strains.

	D2334/11/2/13/CI	M95169 GI 1	GU393336 GI 2	L14069 GI 3	L18988 GI 4	U29522 GI 5	U29519 GI 6	AY606320 GI 7	JQ964061 GI 8	M99482 GI 9	AF151954 GI 10	JX182775 GI 11	X52084 GI 12	EU914938 GI 13	X87238 GI 14	FJ807932 GI 15	KJ941019 GI 16	AF419315 GI 17	AY296744 GI 18	KC577395 GI 19	AF349621 GI 20	DQ064806 GI 21	KC577382 GI 22	AF093796 GI 23	KF757447 GI 24	EU925393 GI 25	FN182243 GI 26	GU301925 GI 27	KX640829 GI 28	KY407556 GI 29	MN696789 GI 30	M21971 GII	U29450 GIII	U77298 GIV	DQ059618 GV	GQ265948 GVI	MH924835 GVII	AY789942 GVIII
D2334/11/2/13/CI																																						
M95169 GI 1	78,0																																					
GU393336 GI 2	79,2	83,4																																				
L14069 GI 3	79,2	84,4	86,8																																			
L18988 GI 4	78,4	79,9	83,7	83,1																																		
U29522 GI 5	78,4	83,5	82,5	82,0	81,6																																	
U29519 GI 6	78,8	84,6	83,1	84,7	82,4	84,9																																
AY606320 GI 7	76,9	83,5	79,2	78,8	78,0	81,6	80,0																															
JQ964061 GI 8	80,1	83,3	85,9	88,2	84,0	82,3	82,7	80,1																														
M99482 GI 9	78,6	79,9	81,9	83,3	83,0	82,1	81,7	78,5	86,1																													
AF151954 GI 10	78,0	83,3	83,3	83,1	81,4	86,5	86,9	80,4	81,4	81,1																												
JX182775 GI 11	79,4	80,4	80,5	81,6	80,5	82,1	82,8	78,3	81,3	80,5	81,3																											
X52084 GI 12	78,7	81,2	81,0	81,1	80,4	83,4	83,7	79,7	81,8	80,5	82,3	82,7																										
EU914938 GI 13	77,6	79,3	77,7	78,5	78,3	79,7	79,4	79,5	78,4	78,8	80,3	78,7	79,0																									
X87238 GI 14	78,8	79,3	79,9	80,3	80,2	80,6	81,3	78,5	80,6	79,7	80,4	81,0	80,6	80,1																								
FJ807932 GI 15	77,7	80,2	80,9	80,4	82,1	83,7	82,0	79,7	81,4	82,9	82,5	80,9	81,3	78,0	81,1																							
KJ941019 GI 16	78,5	82,1	81,4	81,4	80,2	81,8	82,7	78,3	81,7	79,4	81,7	80,3	81,9	79,6	79,8	79,8																						
AF419315 GI 17	78,7	80,5	81,3	80,9	80,6	84,8	81,8	79,0	80,9	83,5	83,6	80,5	81,1	77,6	79,9	84,7	78,9																					
AY296744 GI 18	78,8	80,9	80,9	80,7	81,2	84,7	81,8	79,4	81,6	82,8	82,3	81,8	81,0	78,5	80,9	86,3	80,3	86,6																				
KC577395 GI 19	75,8	78,9	77,6	77,1	77,7	79,8	77,9	81,4	77,6	78,0	78,6	77,7	79,1	79,2	78,5	78,1	78,0	77,2	78,3																			
AF349621 GI 20	77,8	81,3	83,0	83,5	82,8	79,8	81,8	79,2	84,4	83,5	80,3	81,9	80,5	78,4	81,1	79,9	80,2	80,9	81,5	77,0																		
DQ064806 GI 21	78,6	79,1	80,4	80,0	79,7	82,0	82,2	79,9	80,9	79,8	81,0	81,0	81,6	85,0	82,8	79,8	82,3	79,5	80,3	79,6	80,8																	
KC577382 GI 22	77,2	81,9	79,7	80,2	80,2	81,8	81,4	81,1	80,8	80,1	80,8	80,2	79,5	79,2	78,7	79,9	79,3	79,8	81,0	83,2	79,1	78,8																
AF093796 GI 23	78,9	81,0	80,3	80,3	80,2	82,0	82,5	80,3	81,2	80,2	81,9	82,3	84,4	81,4	80,9	81,6	84,0	80,9	81,7	79,5	80,5	82,9	80,6															
KF757447 GI 24	76,1	85,7	80,9	80,3	79,4	81,0	80,3	78,6	81,1	77,9	79,5	78,3	78,7	77,3	76,1	78,0	80,2	77,1	79,3	77,8	77,3	76,1	80,4	79,0														
EU925393 GI 25	76,4	82,3	76,8	77,6	77,5	80,6	79,5	81,6	77,8	77,0	78,5	78,3	78,2	77,6	77,1	78,7	76,5	78,8	79,7	80,0	77,2	78,0	80,8	79,0	78,4													
FN182243 GI 26	80,1	79,7	77,4	77,6	77,1	78,3	79,0	78,7	77,1	77,1	78,1	78,6	78,0	78,0	78,0	77,6	76,2	76,8	77,0	78,3	77,3	77,9	79,0	78,5	76,9	77,1												
GU301925 GI 27	79,4	81,0	82,8	82,1	83,3	85,2	82,9	80,4	83,3	84,8	83,7	80,2	80,9	78,5	81,4	85,0	80,2	86,9	86,0	78,7	81,5	81,1	81,3	81,0	78,0	78,4	77,6											
KX640829 GI 28	76,5	80,3	77,4	76,6	77,6	79,7	78,3	81,1	78,0	78,1	79,3	78,0	79,1	78,6	77,8	79,5	78,6	78,2	79,4	88,3	76,8	79,0	83,5	79,9	79,0	81,3	77,1	79,1										
KY407556 GI 29	77,6	81,1	78,3	78,7	78,5	80,7	79,5	82,1	79,3	78,4	79,6	78,7	80,2	78,5	78,2	78,8	79,2	78,0	79,9	85,4	77,7	78,9	87,4	80,1	79,6	80,4	78,0	79,9	88,5									
MN696789 GI 30	77,0	79,7	78,6	80,1	80,2	80,2	81,4	77,7	79,9	79,1	79,9	79,3	80,1	78,9	83,6	79,9	79,2	80,0	79,8	77,1	79,5	79,9	77,8	80,6	77,8	76,2	78,4	81,0	76,5	77,6								
M21971 GII	60,9	61,5	61,1	61,2	62,1	61,4	60,2	62,9	61,6	62,5	61,0	61,4	60,6	61,3	61,6	61,7	60,0	60,5	63,4	60,0	62,0	62,5	60,8	61,6	61,8	61,7	61,6	61,5	61,7	61,6	60,5							
U29450 GIII	65,3	65,0	64,7	64,6	66,7	65,3	65,9	65,3	66,2	66,2	65,7	64,5	65,0	65,0	65,2	65,9	64,7	65,7	66,6	65,4	65,2	66,1	66,6	64,7	64,5	65,5	64,3	65,9	66,0	65,3	65,7	59,7						
U77298 GIV	61,4	63,8	61,8	61,4	62,6	62,7	62,0	63,1	62,1	62,6	62,2	62,1	62,9	61,6	61,6	63,3	60,9	61,2	63,3	61,2	61,5	62,1	62,4	63,4	62,6	62,6	62,1	61,5	62,8	62,0	60,8	66,6	58,8					
DQ059618 GV	67,6	69,1	69,6	69,3	69,2	69,5	69,8	69,8	70,1	70,2	70,0	69,7	69,5	69,7	69,2	69,2	68,9	68,6	70,6	69,7	69,4	69,7	70,8	69,3	68,5	68,4	67,6	69,9	69,0	70,2	68,3	60,2	64,7	60,4				
GQ265948 GVI	66,9	66,5	67,0	66,5	66,5	68,2	67,3	65,7	68,0	68,1	68,1	66,2	67,3	66,6	66,9	68,5	66,1	69,1	67,7	67,4	67,6	66,1	67,0	66,0	66,1	65,7	66,6	68,5	67,5	67,4	66,3	60,4	70,0	60,0	66,9			
MH924835 GVII	68,3	71,6	70,5	71,3	71,2	71,6	71,2	70,4	71,1	72,1	71,3	69,8	70,7	70,4	71,1	71,3	70,7	70,4	71,7	71,6	71,7	70,7	72,0	70,7	70,9	69,6	69,4	72,6	71,7	71,6	69,5	60,4	62,6	62,6	67,3	65,0		
AY789942 GVIII	60,4	62,1	62,1	61,2	61,7	62,7	61,1	61,5	62,6	62,6	62,7	61,0	61,5	60,3	61,6	62,2	62,5	61,5	62,8	62,7	60,6	60,6	62,3	61,5	61,7	61,4	60,9	62,0	61,6	61,9	61,3	60,1	59,1	62,2	60,0	60,2	61,4	

Table S2. Pairwise amino acid identity matrix of the S1 genomic region between the reference strains.

	D2334/11/2/13/CI	M95169 GI 1	GU393336 GI 2	L14069 GI 3	L18988 GI 4	U29522 GI 5	U29519 GI 6	AY606320 GI 7	JQ964061 GI 8	M99482 GI 9	AF151954 GI 10	JX182775 GI 11	X52084 GI 12	EU914938 GI 13	X87238 GI 14	FJ807932 GI 15	KJ941019 GI 16	AF419315 GI 17	AY296744 GI 18	KC577395 GI 19	AF349621 GI 20	DQ064806 GI 21	KC577382 GI 22	AF093796 GI 23	KF757447 GI 24	EU925393 GI 25	FN182243 GI 26	GU301925 GI 27	KX640829 GI 28	KY407556 GI 29	MN696789 GI 30	M21971 GII	U29450 GIII	U77298 GIV	DQ059618 GV	GQ265948 GVI	MH924835 GVII	AY789942 GVIII
D2334/11/2/13/CI																																						
M95169 GI 1	75,8																																					
GU393336 GI 2	78,2	78,4																																				
L14069 GI 3	78,0	80,1	82,7																																			
L18988 GI 4	74,5	75,2	79,0	77,3																																		
U29522 GI 5	78,8	82,5	81,2	79,7	81,2																																	
U29519 GI 6	77,5	80,6	81,2	81,4	78,0	82,7																																
AY606320 GI 7	75,6	82,1	77,1	78,0	76,2	81,9	77,3																															
JQ964061 GI 8	77,8	79,7	82,1	85,5	78,6	81,9	79,9	79,7																														
M99482 GI 9	77,8	78,4	79,7	81,2	80,1	84,0	80,8	78,4	82,9																													
AF151954 GI 10	77,8	79,3	81,2	79,7	77,8	86,0	85,1	78,6	79,5	80,8																												
JX182775 GI 11	79,3	79,7	79,5	80,1	77,8	84,0	81,2	79,5	79,7	81,2	81,6																											
X52084 GI 12	79,9	79,7	79,5	79,7	78,0	82,1	83,2	81,2	80,1	82,1	81,9	83,2																										
EU914938 GI 13	77,1	77,3	75,6	78,0	73,4	78,0	77,3	78,0	78,2	78,4	77,3	78,0	80,3																									
X87238 GI 14	79,9	78,6	79,3	78,6	78,4	84,2	81,4	79,0	79,5	80,8	81,4	81,4	81,9	78,0																								
FJ807932 GI 15	75,8	76,0	77,3	76,9	77,1	83,8	78,4	77,5	79,5	81,0	79,5	79,9	80,3	76,5	81,9																							
KJ941019 GI 16	78,4	79,7	79,5	79,5	78,2	83,2	80,6	78,6	81,0	79,9	80,6	80,6	82,9	78,0	79,5	78,8																						
AF419315 GI 17	77,3	77,8	78,4	78,6	78,0	86,4	80,3	78,4	78,6	81,2	85,5	81,0	81,6	76,7	80,8	82,5	77,8																					
AY296744 GI 18	77,8	80,1	79,5	79,5	78,8	87,5	81,4	79,0	79,7	82,5	82,9	83,2	81,4	76,7	82,3	82,5	79,5	86,2																				
KC577395 GI 19	75,8	78,8	76,5	76,7	74,9	79,0	76,5	81,9	77,5	77,5	76,9	77,8	80,6	80,6	78,4	77,1	78,6	75,6	77,8																			
AF349621 GI 20	75,8	77,5	80,1	81,4	78,2	81,0	80,3	77,5	81,6	82,5	79,9	81,2	79,7	75,2	80,6	78,0	78,8	79,9	81,0	75,8																		
DQ064806 GI 21	78,6	76,9	79,7	79,5	77,8	82,7	80,3	79,7	80,1	81,0	81,0	81,6	83,2	82,5	81,6	77,5	82,1	79,9	79,7	79,7	81,2																	
KC577382 GI 22	74,3	80,1	77,3	77,3	75,6	81,4	78,4	79,9	78,4	77,1	79,0	77,8	78,2	77,1	78,4	77,1	79,3	77,3	78,0	84,2	75,6	77,8																
AF093796 GI 23	78,6	78,0	79,0	79,9	78,0	83,2	81,0	81,2	80,3	80,8	81,0	81,4	84,7	80,6	82,3	80,8	84,4	80,1	80,3	81,4	80,1	83,4	81,4															
KF757447 GI 24	73,7	81,2	74,7	75,6	73,4	78,0	74,5	75,4	76,7	74,5	73,7	75,6	75,4	72,1	73,4	73,0	76,7	73,0	76,7	75,4	72,1	73,2	76,2	76,2														
EU925393 GI 25	75,4	81,2	74,3	76,9	73,9	78,8	76,2	81,2	76,0	75,2	76,2	77,3	77,1	75,4	76,5	75,8	76,2	76,5	77,3	81,0	75,2	76,0	79,9	77,5	76,0													
FN182243 GI 26	78,2	77,5	74,3	74,1	72,6	76,7	74,5	77,5	74,5	74,3	73,9	76,0	76,9	75,6	76,7	74,7	75,4	73,4	73,9	78,6	74,5	75,4	77,1	76,9	73,9	74,5												
GU301925 GI 27	78,6	80,1	80,8	81,2	80,6	87,0	81,9	78,6	81,9	84,4	83,4	81,0	82,9	77,3	82,1	82,7	81,6	86,0	85,3	77,5	82,7	81,4	79,5	82,9	74,5	76,7	73,9											
KX640829 GI 28	74,9	79,0	74,9	74,5	73,0	79,7	75,6	80,6	75,8	76,7	76,7	76,7	78,4	78,2	76,0	76,9	77,8	76,7	78,8	89,6	74,3	79,0	83,4	80,1	76,5	81,4	76,2	78,0										
KY407556 GI 29	76,2	80,8	74,9	76,7	75,4	80,1	76,7	81,6	77,8	78,0	77,8	78,6	80,6	78,6	76,9	76,7	80,6	76,0	78,4	86,0	75,6	78,4	87,5	80,8	77,5	81,0	76,9	77,5	87,9									
MN696789 GI 30	76,0	77,5	76,7	78,6	76,5	81,2	78,4	79,0	78,8	79,7	78,6	79,3	80,1	77,1	83,4	78,2	78,6	79,3	79,9	77,8	77,8	79,0	76,9	80,3	74,7	73,7	76,9	81,4	75,8	76,9								
M21971 GII	52,1	53,8	51,4	52,1	52,7	53,3	51,6	53,8	52,3	53,3	51,2	53,1	51,6	51,4	54,6	53,1	51,6	51,6	54,9	52,1	53,1	52,5	51,2	53,8	52,9	52,7	51,4	51,8	53,6	53,6	52,1							
U29450 GIII	57,5	58,7	59,6	58,3	57,9	58,5	60,0	59,8	60,3	58,1	57,2	58,7	59,0	58,1	60,7	57,7	57,7	56,8	60,3	58,3	58,3	59,0	59,4	59,2	56,6	56,8	56,2	58,7	57,7	58,3	60,3	49,9						
U77298 GIV	50,8	52,9	49,9	50,1	51,8	51,8	51,6	51,4	52,7	51,8	49,7	49,0	51,6	48,6	52,3	50,1	50,1	49,7	51,6	50,3	51,4	50,3	51,2	52,5	52,3	51,0	50,8	51,4	52,1	51,8	50,8	60,3	47,1					
DQ059618 GV	65,7	63,9	64,8	66,5	65,4	67,0	64,8	67,4	67,8	67,0	65,4	66,5	68,0	67,2	66,1	65,7	66,1	65,4	66,7	67,4	66,3	66,7	68,3	66,5	63,9	65,2	64,1	65,4	68,3	68,9	65,4	51,0	59,0	50,1				
GQ265948 GVI	60,9	60,9	60,7	60,7	60,3	63,1	62,6	59,6	63,5	62,0	62,0	61,8	63,1	61,3	62,2	63,7	60,0	63,5	64,1	62,2	62,0	59,4	60,5	60,7	59,4	59,2	60,7	64,4	62,4	62,0	59,6	52,3	67,4	48,2	59,6			
MH924835 GVII	65,9	70,2	67,0	68,3	68,5	70,8	68,0	68,7	69,3	68,5	68,9	67,0	69,1	67,0	70,6	67,6	70,2	67,2	69,1	68,7	68,7	67,8	69,3	70,6	69,1	68,9	67,6	70,6	68,9	69,8	68,7	52,1	55,7	51,2	63,1	60,7		
AY789942 GVIII	50,8	51,6	52,3	51,4	50,8	52,9	52,7	51,2	52,5	51,2	52,5	50,1	51,6	48,8	51,8	51,0	52,5	51,4	52,5	53,1	50,5	51,0	51,4	52,5	51,8	51,6	51,0	51,8	52,3	52,7	50,5	48,8	48,8	52,1	49,7	51,0	52,7	
